# Listening in: Identifying Considerations for Integrating Complementary Therapy into Oncology Care Across Patient, Clinic, and System Levels—A Case Example of a Digital Meditation Tool

**DOI:** 10.3390/curroncol32120682

**Published:** 2025-12-02

**Authors:** Alexandra Godinho, Sanvitti Dalmia, Krutika Joshi, Christina Seo, Suzi Laj, Francis Cacao, Lisa Lun, Pete Wegier, Punam Rana

**Affiliations:** 1 Humber River Health, Toronto, ON M3M 0B2, Canada; 2 Department of Family and Community Medicine, University of Toronto, Toronto, ON M5G 1V7, Canada; 3 Institute of Health Policy, Management and Evaluation, University of Toronto, Toronto, ON M5G 1V7, Canada

**Keywords:** behavioural design space, human-centered design, complementary medicine, digital meditation tool, clinical decision making

## Abstract

**Purpose**: As cancer treatments and survival rates continue to improve, integrating supportive complementary therapies into oncology practice is increasingly important. Identifying patient- and clinic-level considerations can guide the selection and integration of evidence-based and effective therapies. Using the Behavioural Design Space (BDS), this study illustrates how a design framework can facilitate the identification of patient needs, clinic requirements, and system-level constraints prior to implementing a digital meditation tool (DMT). **Methods**: A cross-sectional survey of cancer patients in active treatment to assess distress, attitudes, barriers, and knowledge of meditation. Descriptive statistics and binary multivariate logistic regressions examined associations between patient characteristics and interest in meditation or using a DMT. Findings were mapped onto the six elements of the BDS framework in consultation with clinic staff. **Results**: Among 148 patients surveyed, 65% had never meditated, yet 42% expressed interest in using a DMT. Greater engagement in stress-coping activities was the strongest predictor of interest in both learning meditation and using a DMT. Female sex increased, while age decreased, the odds of interest in using a DMT. **Conclusions**: Integrating complementary therapies into oncology care requires attention to patient, clinic, and system-level factors. The BDS framework can guide the therapy/tool selection process by highlighting patient needs, potential barriers, and implementation challenges. Future work should focus on operationalizing the BDS into a practical decision-making tool for healthcare providers.

## Simple Summary

As cancer survival rates and treatment improve, there is a growing interest in adding supportive complementary therapies, like meditation, to standard patient care to help individuals cope with their illness. This study explored how digital meditation tools and complementary therapies in general can be introduced into clinics by examining patient needs and barriers, as well as the realities of clinical settings. Cancer patients currently receiving chemotherapy completed self-report questionnaires to assess their stress level, as well as attitudes, interest, and knowledge of meditation. Results from the 148 participants surveyed showed that while most had never meditated, almost half were interested in trying a digital meditation tool. Interest was highest among women, younger patients, and those already engaging in stress-reducing activities such as prayer or listening to music. Combining these findings with clinical staff discussions, we provide a structured way of examining patient, clinic, and hospital-level factors that may impact the integration of supportive therapies into clinical care. Such an approach can ensure that supportive therapies are tailored to patient needs, are successful, and sustainable for clinics.

## 1. Introduction

With advances in cancer detection and treatment improving survival outcomes, cancer is increasingly regarded as both an acute and chronic illness [1]. Survival often extends beyond a year even among individuals with incurable malignancies [2], underscoring the need to address psychological distress alongside physical treatment [3]. This distress can vary from uncertainty about prognosis to fear of disease progression or death, changes to one’s self-concept, and difficulties in social relationships. Such challenges leave patients feeling powerless and overwhelmed, sometimes persisting up to ten years post-treatment [4,5]. Clinically, depression and anxiety affect an estimated 15–49% of patients across different cancer types, stages, and care trajectories. These conditions are associated with more frequent and unplanned medical care (e.g., medical consultations, hospitalizations) [6,7].

Complementary therapies may offer cost- and resource-efficient alternatives to formal psychological services, allowing oncology clinics to integrate more supportive services into routine care. Mindfulness meditation, for example, is a well-established mind-body practice [8] that has been shown to reduce anxiety, depression, fatigue, and pain, while improving sleep and overall quality of life in cancer populations [9,10]. Uptake among cancer patients is high, with up to one third reporting use for supportive care [11], and digital formats now demonstrate comparable efficacy to in-person programs [9,12], making them a viable, low-cost, and scalable solution. However, a narrative review conducted by our team revealed considerable variability in existing program structures, including length, delivery method (teacher vs self-guided), evaluation methodology, and targeted outcomes (e.g., depression, anxiety, quality of life, sleep, and fatigue) [13]. This raises a critical question: how can cancer clinics effectively select digital mindfulness meditation tools or any complementary therapy to offer patients, and how can they anticipate potential implementation challenges?

Aside from selecting an evidence-based program, clinics must also consider whether program features are appropriate for their patients (e.g., duration, intensity, and delivery format), patient characteristics (e.g., readiness to engage and preferences), and organizational factors (e.g., resource allocation, workflows, and policies). Patients often view complementary therapies as self-care, which raises further questions about whether such programs should be formally integrated into clinical care or promoted through educational campaigns [14]. While several implementation science frameworks exist to identify barriers and facilitators and provide strategies for change management—such as the Consolidated Framework for Implementation Science [15] and the Behaviour Change Wheel [16]—most provide limited guidance on *what* to implement, and their strategies largely target healthcare leaders and staff [17]. Alternatively, the Behavioural Design Space (BDS) [18] offers a framework aimed at informing intervention design, which we propose can be adopted by clinics to guide decisions on *what* to implement by anticipating barriers and facilitators of *how* it will be implemented. The BDS outlines six key design elements—cognition, motivation, ability, timing, physical context, and social context—that together shape a person’s capacity and willingness to engage with health-related interventions. Applied to oncology care, this framework can help ensure programs are both evidence-informed and responsive to patient needs.

As part of ongoing work on the feasibility of co-creating a digital meditation tool (DMT) for oncology patients [19], we conducted a cross-sectional survey of oncology patients’ attitudes, knowledge, and experience with meditation. This study offered a unique opportunity to identify the specific needs of our cancer patients, facilitate meaningful discussions with clinic staff, and map our findings onto the BDS framework to guide the future design and/or selection of a DMT. The aim of this study was to identify patient- and clinic-level considerations for integrating a DMT into patient care, while providing a case example to guide other clinics in selecting, designing, or integrating complementary therapies into clinical care. The original survey findings are also presented here.

## 2. Materials and Methods

The study protocol has been published elsewhere as part of a larger study understanding patient and oncologist perceptions of meditation [19]. Briefly, the trial was a single-blinded cross-sectional survey.

### 2.1. Recruitment and Study Design

Participants were recruited from the Humber River Health Cancer Clinic during chemotherapy treatment appointments from August to December 2023. Although the clinic serves patients undergoing chemotherapy, radiation, surgery, and follow-up care, this study used purposive sampling to approach all patients currently receiving chemotherapy, representing a consecutive sample of this subgroup. Eligibility criteria included being English-speaking, 18 or older, and currently receiving treatment for cancer. Individuals with cognitive impairments, psychiatric illness, or those in visible distress were not eligible. Patients were approached in person about their interest in completing a survey about well-being and strategies for coping with potential stress and/or anxiety during their cancer journey. Eligible patients were asked to complete an online consent form and then redirected to an online survey about their general well-being, knowledge, perceptions, and experience with meditation. Participants were debriefed about the true aims of the study (i.e., to understand patient perspectives toward meditation with the aim of developing a DMT) post-study completion and were provided with a meditation handout (Supplement File S1) and a $5 gift card honorarium. Ethics approval for the study was provided by Veritas IRB.

### 2.2. Survey Measures

The survey assessed a variety of demographic and clinical characteristics via self-report, including age, sex at birth, relationship status, education level, employment status, household income, religion, country of birth, type/stage of cancer, and time since diagnosis. In addition, the survey also included measures of psychological well-being: anxiety (General Anxiety Disorder: GAD scale) [20], stress (Perceived Stress Scale: PSS) [21], and mindfulness (Cognitive and Affective Mindfulness Scale–Revised: CAMS-R) [22]. Participant knowledge of meditation was assessed using the general perceptions of meditation [23] questions developed by Russell et al., while barriers to meditating were assessed using the revised version of the Determinants of Meditation Practice Inventory (DMPI) [24]. For a further description of the validity and applicability of these tools to cancer samples, please see protocol [19].

### 2.3. Experience with Meditation

A series of questions, adapted from previous literature [23], were developed to assess participants’ current and past meditation practices (see Supplement File S2). These questions underwent review by the research team and oncology clinic staff to ensure clarity, relevance, and appropriateness for the patient population. As such, a formal pilot was not conducted. One question captured activities to cope with stress (including meditation), and for meditators, additional items addressed the type of practice, resources used, and the frequency of practice post-diagnosis. Participants were asked to use a 7-point Likert-type scale to rate their experience in learning or practicing meditation and perceived benefits. Interest in DMT and willingness to participate in a development focus group were assessed using yes/no questions.

### 2.4. Sample Size

Based on the number of covariates/factors (k = 6) planned for a logistic regression and a lower estimate of the proportion of the positive outcome variable (40%; v = 0.4), the minimum number of cases required was calculated by the formula (N = 10 k/v), resulting in the need to recruit a sample of 150 participants.

### 2.5. Data Analysis

A series of descriptive analyses was used to examine general perceptions and practice trends of meditation, as well as barriers for participants without prior meditation experience. To understand associations between participant characteristics and interest in using a DMT vs. interest in learning about meditation, two separate binary multivariate logistic regressions were used. The outcome variables for the models were interest in using a DMT and interest in learning more about mediation. Interest in learning more about meditation was recoded as a binary yes/no variable, by grouping responses of 5 or more on the Likert-type scale (1-Not Interested at all to 7-Extremely Interested) as yes and 4 or less as no. Key predictor variables were: age, sex, number of activities engaged in to cope with stress (meditation responses were excluded), stress levels (PSS scores), anxiety (GAD scores), and mindfulness (CAMS-R scores). Predictor variables were included in each model as per our pre-specified analysis plan based on theoretical relevance [19]. Multi-collinearity was assessed by calculating the variance inflation factor (VIF) for each variable, and any variable with a VIF value ≥ 5 was removed. All analyses were conducted using Python 3.11.

Following quantitative analyses, survey findings were organized within the Behavioural Design Space (BDS) framework [18] to explore how individual, contextual, and system-level factors could inform the integration of a complementary therapy into oncology care, using a DMT as a case example. This mapping was informed by descriptive trends and existing literature and was completed in consultation with oncology clinic staff, which included a meditation expert.

## 3. Results

In total, 325 patients were approached: 99 declined to participate, 43 were not eligible to participate, and 33 provided consent but did not complete the survey. Overall, 150 consented and completed the survey; however, only 148 patients were retained for analyses. Two participants were excluded (survey completed by a family member). See Table 1 for demographic and clinical characteristics of the sample.

### 3.1. Experience and Interest in Meditation

**Figure 1:**
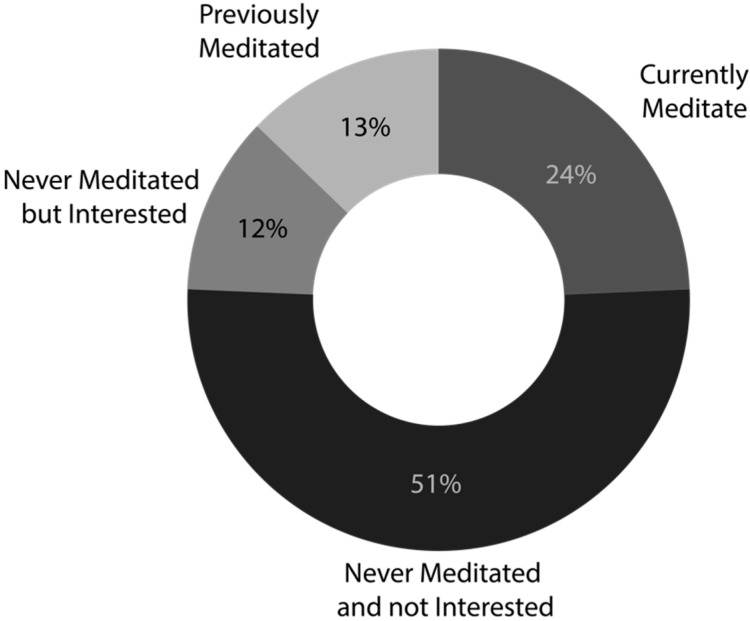
Interest in and practice of meditation across study participants.

To cope with stress/anxiety, participants commonly reported: listening to music (66%), prayer (62%), leisure activities (60%), physical activity (56%), breathing exercises (35%), and meditation (14%). Nearly one quarter (24%) of participants reported practicing meditation in general (an additional 13% reported meditating in the past), of which 44% reported that they currently meditate every day and 31% reported experience with meditation of 10+ years. Overall, 63% of the sample had never meditated, of which 18% were interested in learning about meditation; see Figure 1 for a breakdown of experience and interest in meditation for the whole sample. Lastly, 42% (62/148) of the sample were interested in a DMT, regardless of meditation experience, of which 32 (52%) were interested in participating in a focus group to assist in the development of an online meditation tool.

Current/previous meditators (n = 55) reported that the most common forms of meditation practiced were mindful meditation (58%), guided meditation (33%), and loving kindness meditation (22%). In addition, the tools used for meditating included: music (47%), something to sit on (20%), incense/candles (18%), and digital meditation apps (44%). When examining changes in meditation post cancer diagnosis, 28% of current meditators reported increasing their frequency of practice post diagnosis. Of the 39% who started meditating after their cancer diagnosis, 71% reported beginning because of their diagnosis.

### 3.2. Predictors of Interest in Meditation and a DMT

Multicollinearity checks did not result in the removal of any covariates. Model 1 revealed that interest in learning about meditation was positively associated with the number of stress-coping activities (OR = 1.33). Model 2 revealed that interest in using a DMT was significantly associated with age, sex, and the number of stress-coping activities: being female (OR = 2.29) and engaging in more activities (OR = 1.44) increased interest, while being older decreased interest (OR = 0.97). The full results for both models are presented in Table 2.

### 3.3. General Knowledge and Perceptions of Meditation

**Figure 2:**
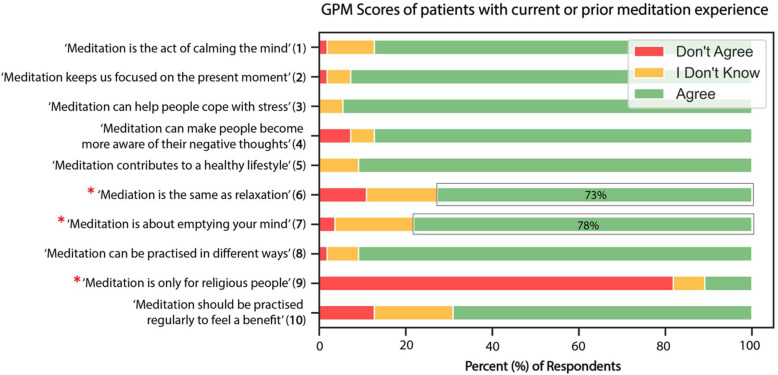

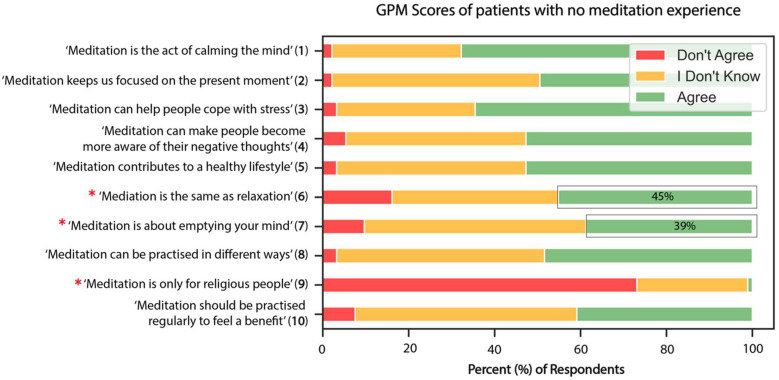
Responses to items on the GPM for those with experience meditating vs. those with no experience. Items with an asterisk represent misconception items. Outlined bars indicate percent of participants who agree with the misconception.

Participants’ knowledge and perceptions of meditation varied across the sample, with an average score of 4.2 (SD = 2.6) on the GPM, out of a possible 10 points. Compared to those with meditation experience (GPM mean = 5.2, SD = 1.7), participants who had never meditated had significantly less overall knowledge of meditation (GPM mean = 3.6, SD = 2.9), t(146) = 3.7, *p* < 0.001. A distribution of participant responses for each item of the GPM for those who have experience with meditation vs. no meditation experience is presented in Figure 2. Across the entire sample, more than half believed that meditation is the same as relaxation (55%) and about emptying the mind (53%); interestingly, these misconceptions were more commonly believed among those *with* prior experience meditating (73% and 78%, respectively).

### 3.4. Barriers to Meditation

**Figure 3:**
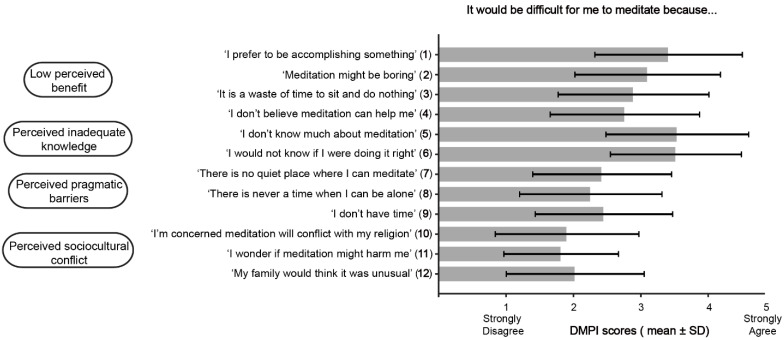
Perceived barriers for meditating among all participants.

**Figure 4:**
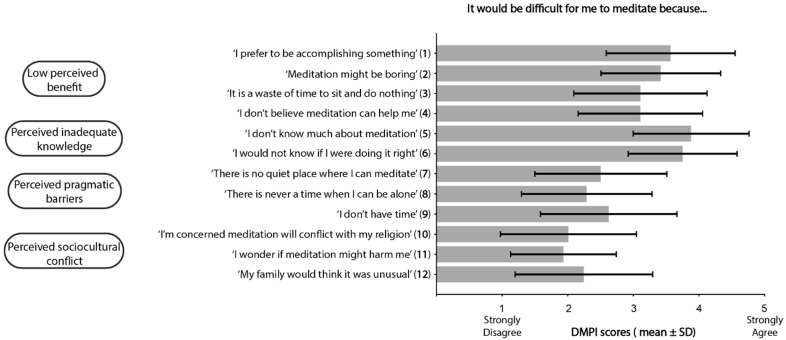
Perceived barriers for meditating among participants with no experience meditating.

Participants reported few barriers to meditation (Figure 3). However, two categories—low-perceived benefit and inadequate knowledge—had items with mean ratings of 3 or higher, at least moderate endorsement of a barrier. For low-perceived benefit barriers, participants endorsed Item 1 (M = 3.4, SD = 1.1) and Item 2 (M = 3.1, SD = 1.1). For perceived inadequate knowledge barriers, the highest-rated items were Item 5 (M = 3.5, SD = 1.1) and Item 6 (M = 3.5, SD = 1.0). Among participants who do not currently, or have never meditated, all low-perceived benefit barriers were endorsed with ratings of 3 or higher (Figure 4); Item 1 (M = 3.6, SD = 1.0) and Item 2 (M = 3.4, SD = 0.9), Item 3 (M = 3.1, SD = 1.0), and Item 4 (M = 3.1, SD = 0.9). In addition, perceived inadequate knowledge barriers were more strongly endorsed compared to the entire sample; Item 5 (M = 3.9, SD = 0.9) and Item 6 (M = 3.9, SD = 0.9).

## 4. Discussion

**Figure 5:**
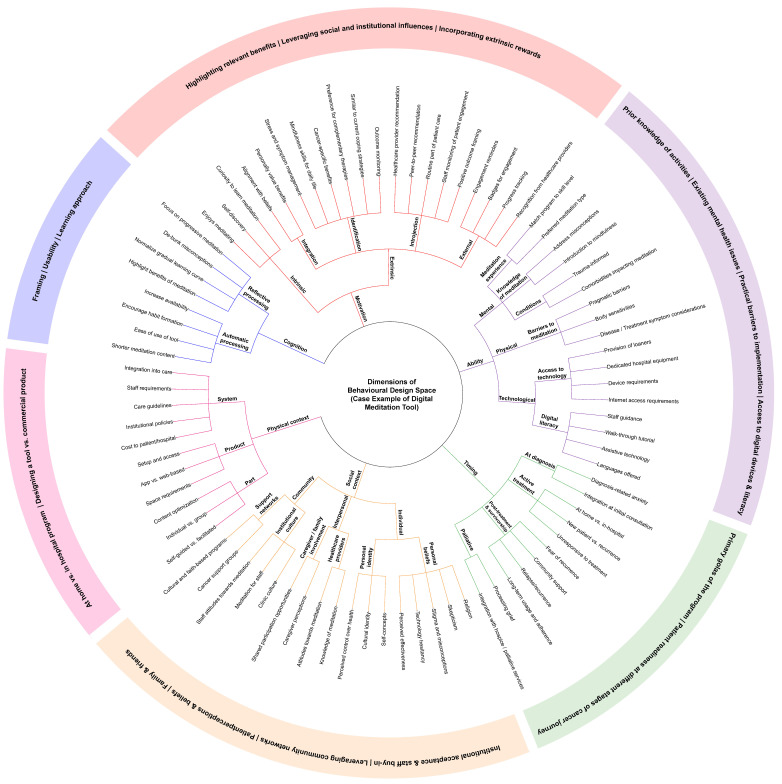
A mapping of study findings and clinical discussions onto the Behavioural Design Space framework.

This study explored knowledge, interest, and general perceptions of meditation among cancer patients in active treatment, with the aim of understanding a specific patient group for implementing a digital meditation platform. Our findings, alongside oncology staff consultations, provide a case example of how the BDS framework can be applied to inform the selection and integration of a complementary therapy into routine care. The following discussion is organized by the six design elements of the BDS and includes recommendations for clinics seeking to integrate supportive interventions. A detailed mapping of findings and clinical discussions onto the BDS framework is presented in Figure 5.

### 4.1. Cognition

Dual-process theories of behavior change distinguish between *automatic processing*—fast, low-effort, and unconscious —and *reflective processing*, slower, effortful, and conscious evaluation of benefits and risks [25,26]. During periods of heightened stress, such as a cancer diagnosis, automatic processing often predominates [25,27], potentially limiting engagement with unfamiliar therapies.

Forty-two percent of patients surveyed expressed interest in using a DMT, and 52% were willing to participate in program development—consistent with evidence that approximately 37.7% of cancer patients use complementary therapies [28]. Surprisingly, participants’ stress levels were comparable to general population norms (study sample M = 15.9, general population: M = 12.7–15.2) [29], and mindfulness scores were within the normative range for cancer populations (study sample: M = 29.7, other cancer samples M = 22.8–29.2) [30,31,32]. These data suggest that some patients have sufficient cognitive and emotional resources to engage reflectively with a mindfulness intervention, especially those willing to co-develop the program. Conversely, patients experiencing acute distress and/or complex treatment burdens, who may not have been surveyed, may benefit more from tools designed for intuitive, low-effort automatic processing.

Three cognitive considerations are recommended:

*Framing*—Highlight benefits, address misconceptions, and emphasize ease of use.

*Usability*—Intuitive and straightforward interface, minimizing barriers to initial and sustained use.

*Learning approach*—Support habit formation and progressive knowledge acquisition.

### 4.2. Motivation

Self-determination theory (SDT) proposes that motivation exists along a continuum, from external regulation (e.g., rewards) to more autonomous forms of introjection (e.g., approval from others), identification (e.g., valuing benefits), integration (e.g., merging behavior with core beliefs), and intrinsic (e.g., genuine interest) [33]. Autonomous forms of motivation are associated with sustained behavioral changes, whereas reliance on external drivers limits long-term adherence [34]. In oncology care, motivation may be impacted by treatment demands, fatigue, or seeing self-care as non-essential [35,36].

While preliminary, patients who engaged in more stress-coping activities were more likely to express interest in both learning about meditation and using a DMT, suggesting stronger autonomous motivation for self-care. Female patients were also more likely to express interest, consistent with evidence that women more frequently adopt complementary therapies [37]. In contrast, older participants showed less interest, potentially reflecting generational differences in attitudes towards complementary care or treatment priorities [37]. Notably, 61% of patients expressed little to no interest in meditation, indicating that strategies relying solely on intrinsic motivation are insufficient.

Therefore, we propose the following motivational considerations:

*Highlighting relevant benefits*—Stress and symptom management, low-risk therapy, and connection to familiar practices.

*Leveraging social and institutional influences*—Normalize meditation through positive support from healthcare providers and peer recommendations.

*Incorporating extrinsic rewards*—Progress tracking, gamification, or recognition from the care team.

### 4.3. Ability

Interventions require patients to have the cognitive and physical capacity for meaningful engagement. For digital interventions, this also includes access to technology and sufficient digital literacy. Cancer-specific barriers and challenges must also be considered, depending on the intervention type.

Twenty-four percent of participants reported practicing meditation: mindful meditation (58%), guided meditation (28%), and loving-kindness (28%). However, prior experience did not reflect an accurate understanding of meditation; meditators were more likely to agree with the misconceptions that meditation is about emptying the mind and is the same as relaxation. It is likely that these individuals conflate the outcomes of meditation (i.e., relaxation) with the process of meditation (i.e., cognitive restructuring), as previous literature has reported similar results [23]. Those without meditation experience reported a lack of knowledge, underscoring the need for introductory content that orients both novices and meditators. Physical symptoms such as fatigue or pain from cancer treatments may also limit engagement [38], while body-focused cues in guided meditation may be distressing for patients with changes to their physical appearance or functioning [39]. Given the high prevalence of symptom-burden cancers in our sample (e.g., gastrointestinal 35%, breast 30%, respiratory 11%), trauma-informed content and adaptable practices are essential.

Although access to technology was not directly assessed, the intervention can be delivered via web browser or available bedside tablets, and with up to 96% of ED patients at this hospital using smartphones, access to technology is sufficient [40]. Nonetheless, lower interest among older adults may reflect digital literacy gaps, warranting further exploration. Lastly, clinic staff emphasized the value of multilingual content, guided staff-support during appointments, and attention to co-occurring mental health concerns that may affect capacity to engage

These findings point to four considerations:

*Prior knowledge of activities*: Assess patients’ current knowledge, misconceptions, skill level, and preferred forms of practice.

*Existing mental health issues*: Consider prior trauma or psychopathology, patients’ comfort/capacity to engage and offer adaptable practices.

*Practical barriers to implementation*: Identify physical challenges (e.g., fatigue, pain, or body sensitivity), environmental conditions, and other logistical concerns.

*Access to digital devices and digital literacy*: Device, internet, language, assistive technology, and supported onboarding.

### 4.4. Timing

Patient readiness to engage in complementary therapies can fluctuate throughout cancer journeys, making timing a critical factor for both well-being and intervention adherence. Intervention delivery can be aligned with diagnosis, active treatment, or survivorship/palliative care, depending on the goals of the intervention, patient stress levels, cognitive load, and distress.

Twenty-eight percent of patients who practiced meditation reported having increased their practice after diagnosis. However, the existing literature suggests that integrating new interventions early in treatment can be challenging due to stress and demanding treatment schedules [41,42]. However, preliminary survey results suggest that patients currently in treatment may be ready, as reported stress levels were low.

Timing decisions should be guided by:

*Primary goals of the program*: Intervention targeting general coping, diagnosis-related anxiety, fear of recurrence, or other.

*Patient readiness at different stages of the cancer journey*: Account for fluctuations in stress, symptom burden, and cancer type/stage.

### 4.5. Physical Context

Evidence suggests that physical space, interfaces, and other environmental considerations can shape behavioral responses and individual well-being in healthcare settings [43,44]. In oncology care, where patients frequently travel from home to clinic environments, it is important to consider the location of the intervention, and how it is integrated into daily routines.

Survey data were complemented by input from clinical staff (including a meditation expert) to identify key physical considerations for use at home and in the clinic. At the *program level*, mindful meditation, music integration, and introductory content are optimal as previously described. At the system level, in-clinic privacy and calming spaces to practice meditation were important, particularly for patients who lack such environments at home. Finally, alignment with institutional policies, workflow capacity, and staff support was seen as essential to long-term feasibility and sustainability.

Overall, two key considerations of physical context are:

*At home* vs. *in-hospital interaction with the program*: Location impacts staffing needs, infrastructure, cost, and compliance with institutional policies (e.g., privacy and care protocols).

*Designing a tool* vs. *a commercial product*: Consider customization, content optimization, delivery format, cost, and timelines.

### 4.6. Social Context

Behavior is rarely an isolated act; it is embedded within interpersonal relationships and broader social structures. Bronfenbrenner’s Ecological Systems Theory [45] explains that multiple nestled social influences (i.e., personal beliefs, close relationships, and cultural norms) affect patient experiences. In oncology care, internalized misconceptions, encouragement from loved ones or clinicians, and community attitudes can all impact engagement with interventions like meditation.

Several meditation misconceptions were identified. Over 20% of non-meditators were unsure whether meditation was only for religious people. Educational efforts could emphasize secular, evidence-based benefits and frame meditation in ways that align with familiar practices or norms. Staff also identified technology avoidance as a barrier for some patients, reinforcing the need to offer options that accommodate varying comfort levels with digital tools. At the interpersonal level, caregivers and clinicians were seen as key influencers of patient decisions to engage in meditation, suggesting that shared participation opportunities could boost adherence. At the community level, staff emphasized the value of embedding meditation into clinic culture—through staff resources, shared practices, and connections with patient support networks, including cultural or faith-based groups.

To ensure integration is socially responsive, we recommend considering:

*Institutional acceptance and staff buy-in*: Alignment with clinic priorities and engagement of healthcare staff, addressing staff knowledge gaps or misconceptions.

*Leveraging community networks*: Peer and community support groups can increase patient trust. Embed the program/tool into broader care pathways.

*Patient perceptions and beliefs*: Understand patient views and tailor messaging to align with personal values and practices.

*Family and Friends*: Involve loved ones in the intervention to support patient engagement and caregiver well-being.

### 4.7. Limitations

A key strength of the study was the inclusion of several cancer types, with recruitment not limited to those interested in meditation (i.e., patients were approached about completing a survey on well-being and strategies for coping with potential stress and/or anxiety during their cancer journey). This approach was novel; however, a few limitations should be considered. Only patients actively in treatment were surveyed (a purposive sample of all cancer clinic patients), and furthermore, only 53% of those eligible completed the survey (i.e., 150/282), limiting the generalizability of results to newly diagnosed patients and survivors. Future research should examine patient perceptions, attitudes, and knowledge at different points in the cancer journey. Additionally, because the original study was not designed within the BDS framework, key patient characteristics—such as motivation, readiness, and digital access/literacy—were not directly measured. Therefore, this work is preliminary in providing guidance to clinics in these areas, rather than prescriptive. Future research should examine associations between different BDS design elements to identify additional barriers and clarify patient motivation/readiness.

## 5. Conclusions

As the burden of cancer continues to rise, it is becoming increasingly important to integrate supportive care, including complementary therapies, into routine oncology practice. Given the many challenges that clinics face in implementing interventions effectively, the BDS offers a useful framework to guide program selection. This paper presents a case example of how the BDS can be applied to identify key considerations when integrating a DMT into care. We highlight patient attitudes and knowledge of meditation, alongside recommendations to inform clinical discussions and systems-level planning around complementary therapies. Future work should focus on operationalizing the BDS into a practical decision-making tool for clinics.

## Figures and Tables

**Table 1:** Participant Demographic and Clinical Characteristics.

Variables	Participants (n = 148)
Demographic Characteristics	
Age, mean (SD)	61.7 (12.6)
Female, % (n)	51.4 (76)
Post-secondary education or higher, % (n)	68.2 (101)
$30,000 income or more, % (n) *	67.3 (76)
Married/common law, % (n)	62.8 (93)
Part/Full Time Employed, % (n)	39.9 (59)
Country of Birth, % (n)	
Canada	28.4 (42)
Italy	13.5 (20)
Philippines	10.8 (16)
Jamaica	10.1 (15)
India	4.1 (6)
Other	33.1 (49)
Christian, % (n)	72.3 (107)
Clinical Characteristics	
Primary Cancer Diagnosis, % (n)	
Gastrointestinal cancer	35.1 (52)
Breast cancer	30.4 (45)
Blood cancer	14.9 (22)
Respiratory cancer	9.5 (14)
Genitourinary cancer	8.1 (12)
Other	2.0 (3)
Cancer Stage, % (n)	
I	8.1 (12)
II	14.9 (22)
III	21.6 (32)
IV	24.3 (36)
Unsure/don’t know	31.1 (46)
Time Since Diagnosis, mean months (SD)	26 (40)
First Diagnosis, % (n)	83.1 (123)
PSS ^a^, mean (SD)	15.9 (7.1)
GAD ^b^, mean (SD)	5.4 (5.1)
CAMS ^c^, mean (SD)	29.7 (6.6)

Note. * Missing data for n = 35 (preferred not to disclose). ^a^ PSS = Perceived Stress Scale, ^b^ GAD = Generalized Anxiety Disorder Scale, and ^c^ CAMS = Cognitive and Affective Mindfulness Scale.

**Table 2:** Binomial multivariate logistic regression assessing participant characteristics associated with interest in learning about meditation (Model 1) and interest in a DMT (Model 2).

Variables	ß (SE)	Odds Ratio (CI)	*p*
Model 1: Interest in Learning About Meditation (R^2^ * = 0.16)			
Constant	1.834	0.88	0.94
Age	0.016	0.98 (0.95–1.01)	0.25
PSS ^a^	0.043	0.99 (0.91–1.09)	0.81
GAD ^b^	0.052	1.05 (0.95–1.16)	0.36
CAMS ^c^	0.035	0.97 (0.90–1.03)	0.33
Number of Other Stress Activities	0.13	1.33 (1.03–1.72)	0.03
Female	0.409	2.06 (0.92–4.59)	0.08
Model 2: Interest in a DMT (R^2^ * = 0.23)			
Constant	1.77	0.91	0.96
Age	0.016	0.97 (0.94–1.00)	**0.05**
PSS ^a^	0.041	0.96 (0.88–1.04)	0.30
GAD ^b^	0.052	1.10 (1.00–1.22)	0.06
CAMS ^c^	0.033	1.01 (0.94–1.08)	0.80
Number of Other Stress Activities	0.129	1.44 (1.12–1.86)	<0.01
Female	0.391	2.29 (1.06–4.93)	0.03

Note. Bold denotes *p* < 0.05 * Nagelkerke’s R^2^, ^a^ PSS = Perceived Stress Scale, ^b^ GAD = Generalized Anxiety Disorder Scale, and ^c^ CAMS = Cognitive and Affective Mindfulness Scale.

## Data Availability

Data are available from the corresponding author upon reasonable request.

## Supplementary Materials

The following supporting information can be downloaded at: https://www.mdpi.com/article/10.3390/curroncol32120682/s1, File S1: Meditation Pamphlet for Participants; File S2: Online Patient Survey Questionnaire.
