# Supplementary material for: Listening in: Identifying Considerations for Integrating Complementary Therapy into Oncology Care Across Patient, Clinic, and System Levels—A Case Example of a Digital Meditation Tool
Source: Curr Oncol. 2025 Dec 2;32(12):682. doi: 10.3390/curroncol32120682 (PMC12731991; doi:10.3390/curroncol32120682)

Where can I find out more?

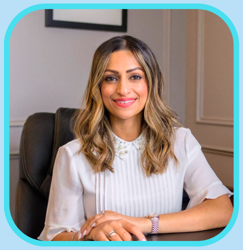

Dr. Punam Rana is a medical oncologist at Humber River Health and certified meditation teacher. She is committed to educating and promoting meditation for patients with cancer and has devoted an entire section of her website to providing patients with the resources and information they need to explore whether or not meditation is right for them.

You can visit her website at **https://drpunamrana.com.**

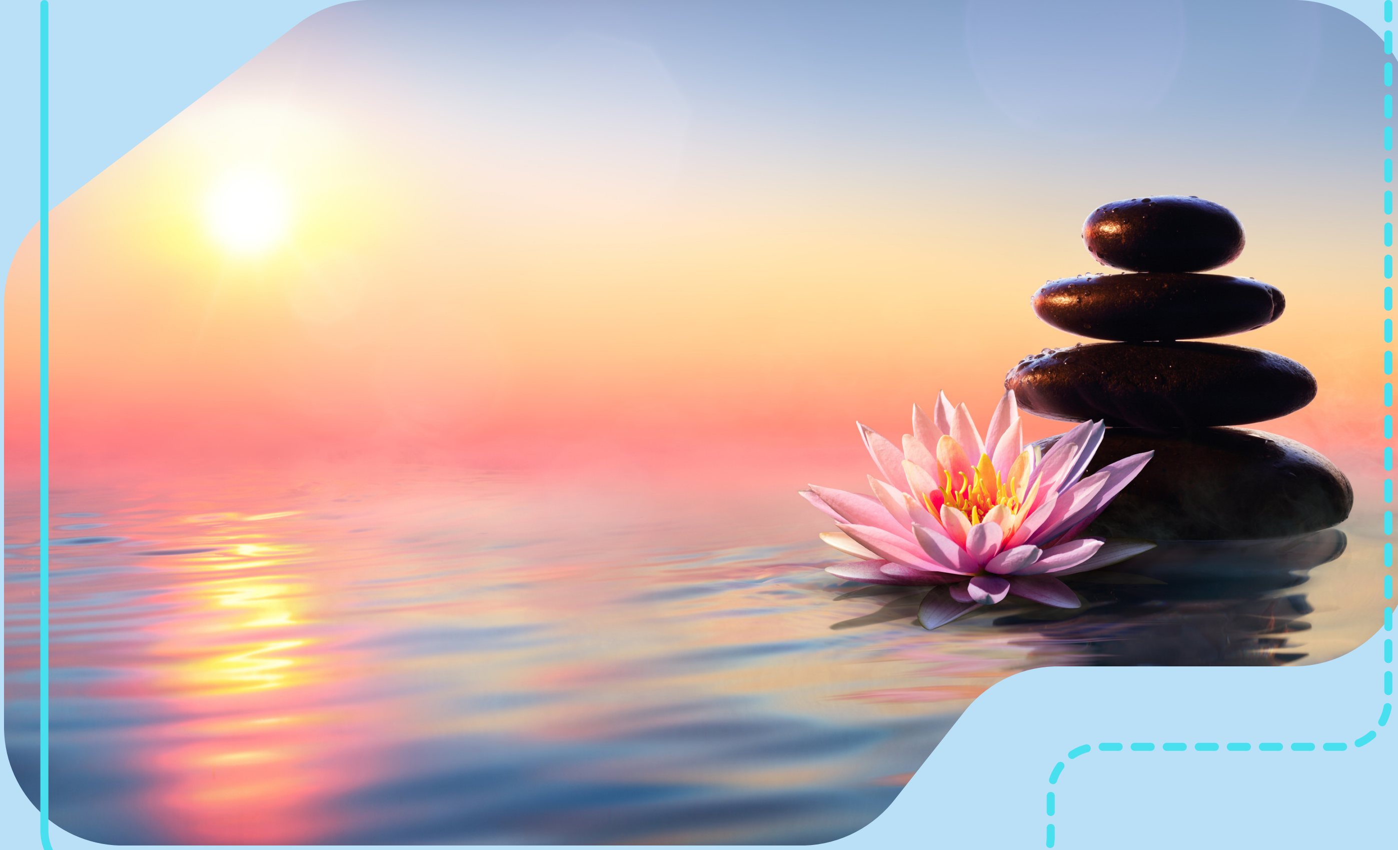

This pamphlet was created by the Humber River Health Research Institute to educate patients with cancer about the ways that meditation may be beneficial to them.

Meditation

A Resource for Patients with Cancer

Humber River Health
Research Institute

What is Meditation?

Meditation refers to a variety of practices that involve focused attention on the present moment and calming the mind.

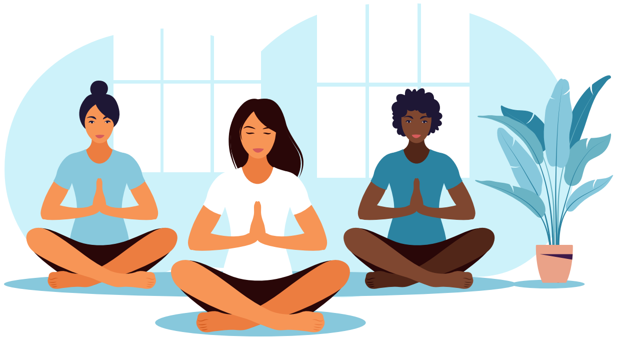

Meditation can take on many different forms and can incorporate a range of techniques including breath, sound, movement, visualization, or music.

How can it benefit me?

For patients with cancer, meditative practices can help to foster calmness, clarity, and overall wellbeing.

Studies have shown that meditation can reduce symptoms such as anxiety, mood disturbance, and depression, and improve overall quality of life.

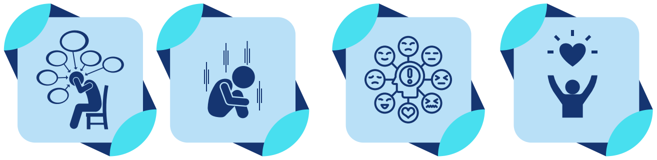

Other studies suggest it can also help those experiencing insomnia, high blood pressure, and chronic pain.

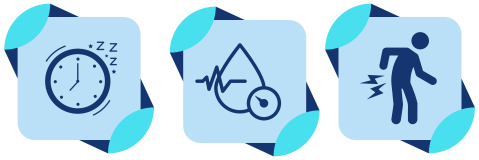

Supplement: Supplementary file 1 [file curroncol-32-00682-s001.zip › curroncol-3939360-supplementary/Supplement File S1.pdf]
