# Supplementary material for: Listening in: Identifying Considerations for Integrating Complementary Therapy into Oncology Care Across Patient, Clinic, and System Levels—A Case Example of a Digital Meditation Tool
Source: Curr Oncol. 2025 Dec 2;32(12):682. doi: 10.3390/curroncol32120682 (PMC12731991; doi:10.3390/curroncol32120682)
Supplement: Supplementary file 1 [file curroncol-32-00682-s001.zip › curroncol-3939360-supplementary/Supplement File S2.pdf]

Online Patient Survey Questionnaire

1. Do you engage with any of the following activities to help with stress / anxiety? (Please check all that apply) (responses below will appear in random order):
   - ❑ Meditation
   - ❑ Mindfulness based stress reduction
   - ❑ Listening to music
   - ❑ Prayers
   - ❑ Breathing exercises
   - ❑ Physical Activity
   - ❑ Leisure Activities
   - ❑ None / I don't do any
   - ❑ Other (Please specify): __________________

*Only ask 2 if meditation or is not selected for 1.

2. Do you engage in ***any*** form of meditation?
   - ❑ Yes
   - ❑ No

*For those that select yes proceed to 3 otherwise ask 2a.

2a. Have you *ever* meditated in the past?
- ❑ Yes
- ❑ No

If 2 is yes, or 2a is yes proceed to 3, otherwise skip to 10

3. What type of meditation do (did) you practice? (please select all that apply):
   - ❑ Mindful meditation
   - ❑ Transcendental meditation
   - ❑ Guided meditation
   - ❑ Vipassana meditation
   - ❑ Loving Kindness/Metta meditation
   - ❑ Other (please specify): ____________________

4. What resources do (did) you use to meditate (Select all that apply):
   - ❑ Smartphone App
   - ❑ Book
   - ❑ Website
   - ❑ Virtual group
   - ❑ In-person group
   - ❑ Spiritual Leader
   - ❑ Certified Meditation Teacher
   - ❑ Other (please specify): _____________

5. Do (Did) you use any of the following tools to help you meditate? (Select all that apply):
   - ❑ Incense / Candles
   - ❑ Mat / Cusion / Seat / Blanket
   - ❑ Bells / Chimes / Singing Bowl
   - ❑ Malas (i.e. Beaded Necklace)
   - ❑ Mandalas (i.e. geometric configuration of symbols)
   - ❑ Prayer Wheel
   - ❑ Music
   - ❑ Crystals
   - ❑ Zen Garden (including desktop sized ones)
   - ❑ I don't use anything
   - ❑ Other (Please specify): _______________

6. How long have (did) you practiced meditation?
   - ❑ Less than 6 months
   - ❑ 6 months – 1 year
   - ❑ 1 – 5 years
   - ❑ 5 – 10 years
   - ❑ 10 + years

6a. Did you start meditating after you received your diagnosis of cancer?
   - ❑ Yes
   - ❑ No

If yes proceed to 6b otherwise skip to 7.

6b. Did you start meditating because of your cancer diagnosis?
   - ❑ Yes
   - ❑ No

7. Has(Did) the frequency of your meditation changed since your cancer diagnosis:
   - ❑ I meditate more often
   - ❑ Stayed the same
   - ❑ I meditate less often

8. How often do (did) you meditate?
   - ❑ Every day
   - ❑ A few times a week
   - ❑ Once a week
   - ❑ 2-3 times a month
   - ❑ Less than once a month

9. Which of the following best describes when you (have) meditate(d)?
   - ❑ I meditate(d) regularly
   - ❑ I meditate(d) after a stressful event
   - ❑ I meditate(d) whenever I remember to

- ❑ I meditate(d) when I am bored
- ❑ I meditate(d) while waiting for an appointment

*For those who currently meditate (Meditation is selected for 1, or yes to 2), options in brackets will be asked.

10. How interested are you in learning (more) about meditation?

Not at all Interested						Extremely Interested
1	2	3	4	5	6	7

11. How interested are you in practicing (continuing) meditation?

Not at all Interested						Extremely Interested
1	2	3	4	5	6	7

12. To what extent do you think meditating (would) help(s) you?

Not at all						A great deal
1	2	3	4	5	6	7
